# Diagnostic accuracy of three-dimensional transvaginal ultrasound for intrauterine adhesions: a systematic review and meta-analysis

**DOI:** 10.3389/fmed.2025.1690719

**Published:** 2025-11-18

**Authors:** Liyin Huang, Zhiping Huang, Bijuan Hu

**Affiliations:** Department of Ultrasound, Ganzhou People’s Hospital, Ganzhou, Jiangxi, China

**Keywords:** intrauterine adhesions, three-dimensional transvaginal ultrasonography, hysteroscopy, diagnostic accuracy, meta-analysis

## Abstract

**Background:**

Intrauterine adhesions (IUA) are a common cause of menstrual abnormalities, infertility, and adverse pregnancy outcomes. Hysteroscopy is the diagnostic gold standard but is invasive and less accessible in some clinical settings. Three-dimensional transvaginal ultrasonography (3D-TVUS) offers a non-invasive alternative; however, its diagnostic accuracy for IUA remains uncertain.

**Objective:**

To systematically evaluate the diagnostic performance of 3D-TVUS for detecting IUA, using hysteroscopy as the reference standard.

**Methods:**

A systematic review and meta-analysis were conducted following PRISMA-DTA guidelines. PubMed, Wiley library, Web of Science, Cochrane Library, and major Chinese databases were searched up to August 1, 2025. Eligible diagnostic accuracy studies reported sensitivity, specificity, or data allowing construction of 2 × 2 tables. Two reviewers independently screened studies, extracted data, and assessed quality using QUADAS-2. A bivariate random-effects model was used to pool sensitivity, specificity, positive and negative likelihood ratios (PLR, NLR), diagnostic odds ratio (DOR), and hierarchical summary receiver operating characteristic (HSROC) curve.

**Results:**

Nine studies involving 2,830 participants were included. The pooled sensitivity was 0.86 (95% CI: 0.83–0.89) and specificity was 0.90 (95% CI: 0.87–0.92). The pooled PLR and NLR were 8.6 (95% CI: 6.2–11.9) and 0.16 (95% CI: 0.13–0.20), respectively. The pooled DOR was 53.2 (95% CI: 34.7–81.4). The HSROC curve yielded an area under the curve (AUC) of 0.94 (95% CI: 0.91–0.96), indicating high diagnostic accuracy. Fagan’s nomogram demonstrated a substantial increase in post-test probability following a positive test result and a marked decrease following a negative result.

**Conclusion:**

3D-TVUS demonstrates high sensitivity, specificity, and overall accuracy for diagnosing intrauterine adhesions, supporting its potential as a reliable, non-invasive first-line diagnostic tool. Further multicenter, prospective studies with standardized imaging protocols are warranted to validate these findings and optimize clinical application.

## 1 Introduction

Intrauterine adhesions (IUA) are pathological intrauterine structural abnormalities characterized by the formation of fibrous bands or adhesions partially or completely obliterating the uterine cavity, resulting from damage to the basal layer of the endometrium (1, 2).

Recent preclinical studies have further elucidated the mechanisms underlying IUA development following endometrial injury, highlighting the critical role of impaired repair and excessive fibrosis. For instance, Genco et al. (3) conducted a prospective laboratory study in rat models with experimentally induced Asherman syndrome (a severe form of IUA), demonstrating that filgrastim (granulocyte-colony stimulating factor) combined with hyaluronic acid promotes endometrial regeneration by upregulating vascular endothelial growth factor (VEGF) expression and reducing fibrotic tissue formation—key processes for restoring the integrity of the endometrial basal layer (4). Similarly, another study by the same research team found that methylprednisolone (a synthetic glucocorticoid) paired with hyaluronic acid mitigates inflammation-driven adhesion progression in rat models, underscoring the importance of targeting inflammatory pathways to prevent irreversible endometrial damage (5). These animal model findings provide valuable insights into the pathophysiology of IUA, reinforcing that inadequate endometrial repair post-injury is a core driver of adhesion formation.

They commonly occur secondary to postpartum or post-abortion curettage, intrauterine infection, or transcervical surgical procedures such as hysteroscopy, and may also develop following the insertion of an intrauterine device or other intrauterine interventions with inadequate endometrial repair (6). In addition, uterine surgical and interventional procedures—most notably myomectomy and uterine artery embolization performed for fibroids—are recognized iatrogenic risk factors for endometrial basal-layer injury and subsequent adhesion formation. Clinically, IUA can lead to hypomenorrhea, amenorrhea, menstrual irregularities, secondary infertility, and recurrent pregnancy loss, and are closely associated with severe obstetric complications including placenta accreta spectrum, retained placenta, and preterm birth (7, 8). Their high recurrence rate and substantial impact on women’s reproductive health make early and accurate diagnosis a critical component of clinical management.

Hysteroscopy is widely regarded as the diagnostic reference standard for IUA, allowing direct visualization of the extent, severity, and location of adhesions, with the advantage of enabling simultaneous operative management (adhesiolysis) when indicated (9, 10). However, hysteroscopy is an invasive procedure that requires appropriate surgical facilities, specialized instrumentation, and anesthesia, making it unsuitable for large-scale screening or frequent postoperative surveillance (11–13). In addition, limited access to equipment and experienced operators remains a challenge in resource-constrained or primary care settings. Therefore, there is a pressing need for a non-invasive, reproducible, and diagnostic reliable imaging modality to improve detection rates and reduce the risk of delayed diagnosis in IUA.

Conventional two-dimensional transvaginal ultrasonography (2D-TVUS) is a commonly used modality for uterine cavity assessment due to its non-invasive nature, cost-effectiveness, and convenience (14, 15). It allows evaluation of uterine morphology, endometrial thickness, and echogenicity in sagittal and transverse planes. However, 2D-TVUS has limited ability to display the coronal plane, detect mild or focal adhesions, and assess the symmetry of the uterine funds and cornual regions. As a result, subtle or localized lesions are often missed, and diagnostic performance is influenced by operator experience, transducer quality, and scanning protocols. In contrast, three-dimensional transvaginal ultrasonography (3D-TVUS) enables volumetric acquisition and multiplanar reconstruction, providing coronal plane images that depict the overall contour of the uterine cavity and the junctional zone (16, 17). When combined with surface rendering, volume contrast imaging, and three-dimensional power Doppler, 3D-TVUS can more comprehensively assess intrauterine bands, endometrial defects, and vascular perfusion (18). The addition of 3D-SIS can further enhance the delineation of lesion boundaries and the measurement of cavity volume.

Although numerous clinical studies have suggested that 3D-TVUS offers superior visualization and diagnostic performance compared with 2D-TVUS, reported accuracy varies considerably across studies, and in some cases, findings are contradictory (19–21). This inconsistency may be attributable to small sample sizes, heterogeneity in study populations, differences in image acquisition and reconstruction techniques, lack of standardized diagnostic thresholds, and inconsistent use of hysteroscopy as the reference standard. To date, no comprehensive quantitative synthesis within a diagnostic test accuracy (DTA) systematic review framework has evaluated the performance of 3D-TVUS for diagnosing IUA. This gap in evidence limits clinicians’ confidence in selecting 3D-TVUS for screening, definitive diagnosis, or postoperative follow-up in different clinical contexts.

In light of these considerations, this study was designed in accordance with the PRISMA-DTA reporting guidelines to conduct systematic review and meta-analysis, using hysteroscopy as the reference standard, to quantitatively assess the sensitivity, specificity, positive and negative likelihood ratios, diagnostic odds ratio, and area under the summary receiver operating characteristic (SROC) curve of 3D-TVUS in diagnosing IUA. Furthermore, the methodological quality and clinical applicability of the included studies will be critically appraised. The findings of this study are expected to provide high-quality evidence for gynecologists and sonographers, clarify the role of 3D-TVUS in the diagnosis and management of IUA, and inform strategies for optimizing patient screening pathways and improving reproductive outcomes.

## 2 Methods

### 2.1 Study design and registration

This study is a systematic review and meta-analysis of DTA, conducted and reported in strict accordance with the Preferred Reporting Items for a Systematic Review and Meta-analysis of Diagnostic Test Accuracy Studies (PRISMA-DTA) statement.

### 2.2 Literature search strategy

Two reviewers independently conducted a comprehensive literature search in PubMed, Wiley library, Web of Science, the Cochrane Library, CNKI, Wanfang Data, and VIP Database. The search covered all records from database inception to 1 August 2025, with no restriction on publication year. The search strategy combined Medical Subject Headings with free-text terms, tailored for each database. English search terms included, but were not limited to, “intrauterine adhesions” OR “Asherman syndrome” AND “three-dimensional transvaginal ultrasonography” OR “3D transvaginal ultrasound” OR “3D-TVUS,” combined with “diagnosis,” “diagnostic accuracy,” “sensitivity,” and “specificity.”

### 2.3 Eligibility criteria

Studies were eligible for inclusion if they met all of the following criteria: (1) Study type: DTA studies, including prospective or retrospective cohort studies, case–control studies, or cross-sectional designs; (2) Population: Women with suspected or confirmed intrauterine adhesions, regardless of etiology, symptom severity, or reproductive history; (3) Index test: 3D-TVUS, with or without adjunctive techniques such as 3D-SIS; (4) Reference standard: Hysteroscopy as the diagnostic gold standard; (5) Outcomes: Sufficient data to construct a 2 × 2 contingency table (true positives, false positives, false negatives, true negatives) or directly report sensitivity and specificity.

Studies were excluded if they met any of the following criteria: (1) Non-human studies or studies using phantom models; (2) Conference abstracts, letters, editorials, reviews, meta-analyses, or case reports without original diagnostic data; (3) Studies lacking a clear reference standard or not using hysteroscopy as the comparator; (4) Incomplete or unusable data even after contacting the corresponding author; (5) Duplicate publications or overlapping patient populations (in which case, the study with the largest sample size or most complete data was retained).

### 2.4 Study selection and data extraction

Two reviewers independently screened and extracted data from the retrieved literature. Titles and abstracts were first reviewed to exclude studies that clearly did not meet the inclusion criteria, followed by full-text assessment of potentially eligible articles to determine final inclusion. Any disagreements during the selection process were resolved through discussion with a third reviewer. Data extraction was conducted using a predesigned standardized form, which included: (1) basic bibliographic information: first author’s name, year of publication, and country/region of study; (2) study characteristics: sample size and participants’ age; and (3) outcome measures: 2 × 2 contingency table data (true positives, false positives, false negatives, and true negatives) that were directly reported or could be derived from the study, or sensitivity and specificity values.

### 2.5 Risk of bias and applicability assessment

The methodological quality of the included studies was assessed using the Quality Assessment of Diagnostic Accuracy Studies 2 (QUADAS-2) tool. This tool comprises four domains: patient selection, index test, reference standard, and flow and timing. In accordance with the predictive context of the present study, certain signaling questions were adapted, such as incorporating the adequacy of follow-up and completeness of outcome reporting into the “flow and timing” domain. For each domain, the risk of bias was rated as “low,” “high” or “unclear” and applicability concerns were rated as “low,” “high” or “unclear” The assessment was independently conducted by two reviewers, and any disagreements were resolved through discussion or consultation with a third reviewer.

### 2.6 Statistical analysis

A bivariate random-effects model was applied to jointly pool sensitivity and specificity, and a SROC curve was constructed to assess the overall performance of three-dimensional transvaginal ultrasonography in diagnosing intrauterine adhesions. Diagnostic odds ratio (DOR), positive likelihood ratio (PLR), negative likelihood ratio (NLR), and area under the curve (AUC) were also calculated and pooled. Heterogeneity was evaluated by plotting 95% confidence and prediction ellipses, calculating the covariance between sensitivity and specificity, and supplementing with the Cochran-Q test and I^2^ statistic for the pooled DOR. Publication bias was assessed using Deeks’ funnel plot and regression test. To evaluate post-test probabilities and clinical utility, Fagan nomograms were generated under varying pre-test probabilities, and likelihood ratio scatter plots were constructed with quadrant-based interpretation. All statistical analyses were conducted using Stata software.

### 2.7 Sensitivity and influence analyses (new)

In addition to the primary bivariate random-effects model, we conducted pre-specified sensitivity analyses to assess robustness: (i) exclusion of any study rated high risk of bias in QUADAS-2; and (ii) leave-one-out analyses, omitting each study in turn. We compared pooled sensitivity, specificity, and HSROC AUC from these analyses with the primary estimates. We also inspected changes in the 95% confidence and prediction regions to judge practical impact.

## 3 Results

### 3.1 Study selection process

**FIGURE 1:**
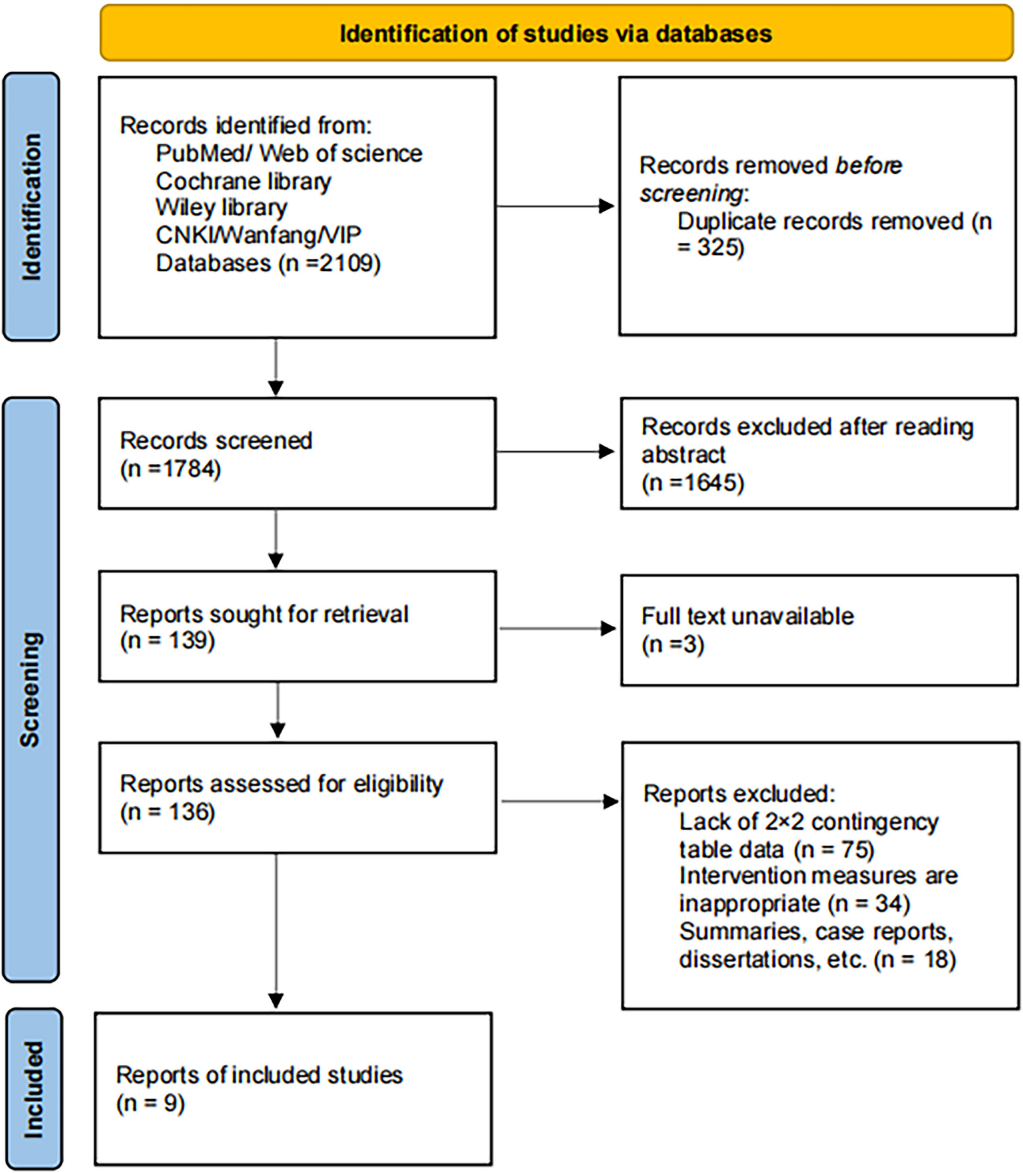
PRISMA flow diagram.

A total of 2,109 records were identified from PubMed, Web of Science, the Cochrane Library, Wiley Library, CNKI, Wanfang, and VIP databases. After removing 325 duplicates using EndNote, 1,784 records remained for screening. Following title and abstract review, 1,645 records were excluded for not meeting the inclusion criteria. The remaining 139 records were sought for full-text retrieval, with 3 excluded due to unavailability of the full text. A total of 136 articles were assessed for eligibility, of which 128 were excluded due to lack of sufficient data to construct a 2 × 2 contingency table (*n* = 75), inappropriate intervention measures (*n* = 34), or being reviews, case reports, dissertations, or other non-original studies (*n* = 18). Ultimately, nine studies (19–27) were included in the quantitative synthesis. The study selection process is presented in Figure 1.

### 3.2 Basic characteristics of the included studies

A total of nine studies were included in this systematic review. All studies used hysteroscopy as the reference standard to evaluate the diagnostic performance of three-dimensional transvaginal ultrasonography (3D-TVUS) for intrauterine adhesions. The AUC values ranged from 0.737 to 0.963, with sensitivity (SEN) ranging from 60.71 to 94.30% and specificity (SPE) ranging from 55.20 to 97.00%. All studies reported raw data that allowed the construction of 2 × 2 contingency tables, including true positives (TP), false positives (FP), false negatives (FN), and true negatives (TN). The detailed characteristics are summarized in Table 1.

**TABLE 1:** Basic characteristics of included studies.

Author (year)	Country/Region	Sample size (n)	Age (years)	AUC	SEN	SPE	Main diagnostic metrics			
							TP	FP	FN	TN
Han, H. 2019 (19)	China	600	27.1 ± 10.5	0.963	94.30%	94.30%	482	5	30	83
Liu, S. 2023 (20)	China	120	24∼39	0.927	93.30%	85.00%	56	9	4	51
Pang, H. 2022 (21)	China	176	32.59 ± 6.42	0.737	85.20%	55.20%	99	27	17	33
Xie, J. 2024 (22)	China	66	29.52 ± 2.77	0.88	74.20%	88.60%	24	4	8	30
Zhao, X. 2025 (23)	China	688	> 20	0.857	85.30%	92.60%	363	19	63	243
Zhao, X. 2022 (24)	China	401	31.4 ± 5.65	0.912	87.50%	97%	139	8	4	250
Zhao, Y. 2024 (25)	China	266	20∼40	0.827	74.80%	83.70%	107	20	36	103
Zhong, L. 2025 (26)	China	112	30.05 ± 3.36	0.837	60.71%	88.39%	81	3	33	25
Zhou, J. 2023 (27)	China	401	31.2 ± 5.59	0.801	75.30%	86.75%	108	34	35	224

### 3.3 Risk of bias and applicability assessment

According to the QUADAS-2 assessment, most studies had a low risk of bias in patient selection, index test, and reference standard domains. Only two studies showed high risk in the selection or flow and timing domains, and one study had an unclear risk in the index test domain. Regarding applicability, all studies were rated as low concern except for one study with high concern in the index test domain (Table 2).

**TABLE 2:** Risk of bias and applicability concerns of included studies according to the QUADAS-2 tool.

Author (year)	Patient selection	Index test	Reference standard	Flow and timing	Applicability: patient selection	Applicability: index test	Applicability: reference standard
Han, H. 2019 (19)	Low	Low	Low	Low	Low	Low	Low
Liu, S. 2023 (20)	Low	Low	Low	Low	Low	Low	Low
Pang, H. 2022 (21)	High	Low	Low	High	Low	Low	Low
Xie, J. 2024 (22)	Low	Low	Low	Low	Low	Low	Low
Zhao, X. 2025 (23)	Low	Low	Low	Low	Low	Low	Low
Zhao, X. 2022 (24)	Low	Unclear	Low	Low	Low	High	Low
Zhao, Y. 2024 (25)	Low	Low	Low	High	Low	Low	Low
Zhong, L. 2025 (26)	High	Low	Low	Low	Low	Low	Low
Zhou, J. 2023 (27)	Low	Low	Low	Low	Low	Low	Low

### 3.4 Combined results of main effects

**FIGURE 2:**
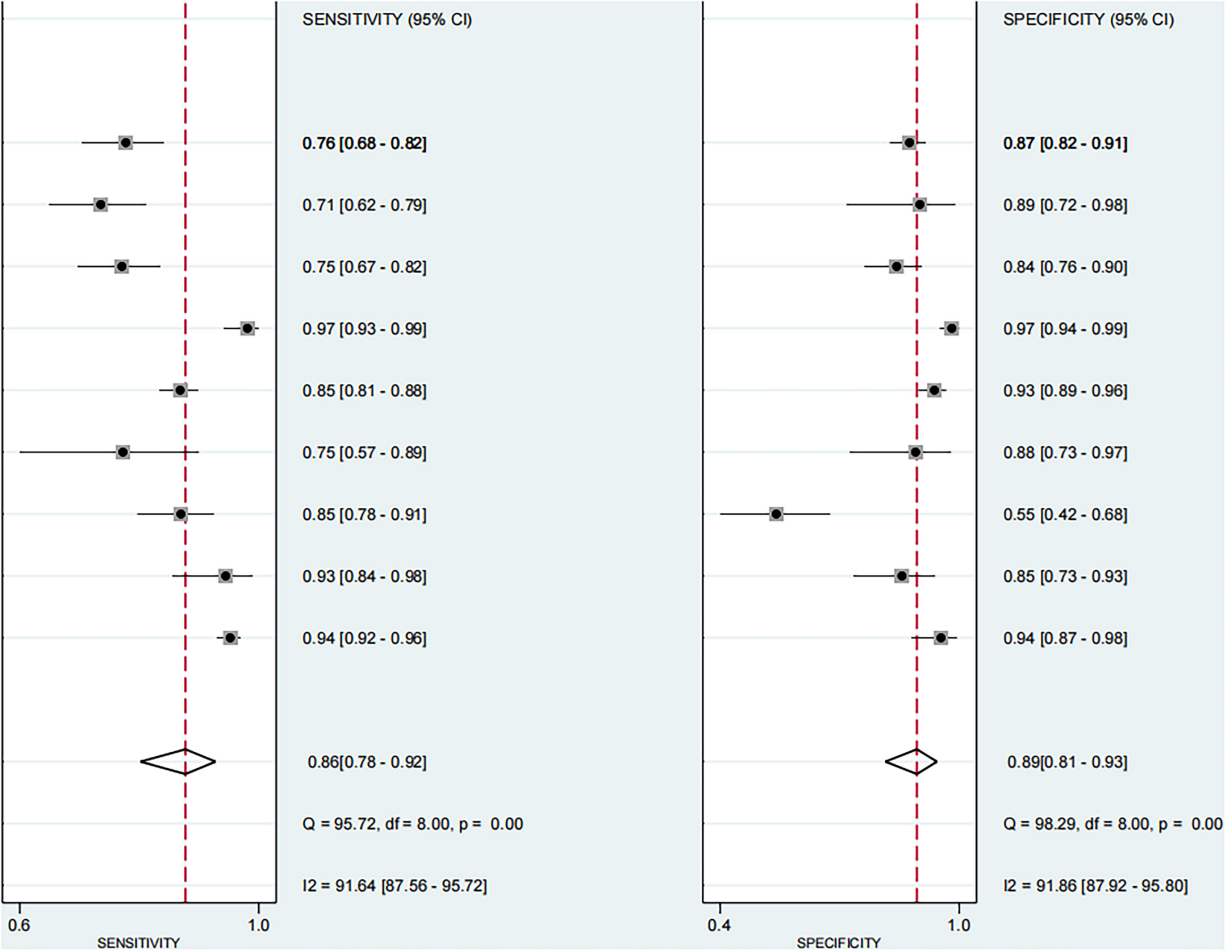
Forest plots of sensitivity and specificity for each included study.

**FIGURE 3:**
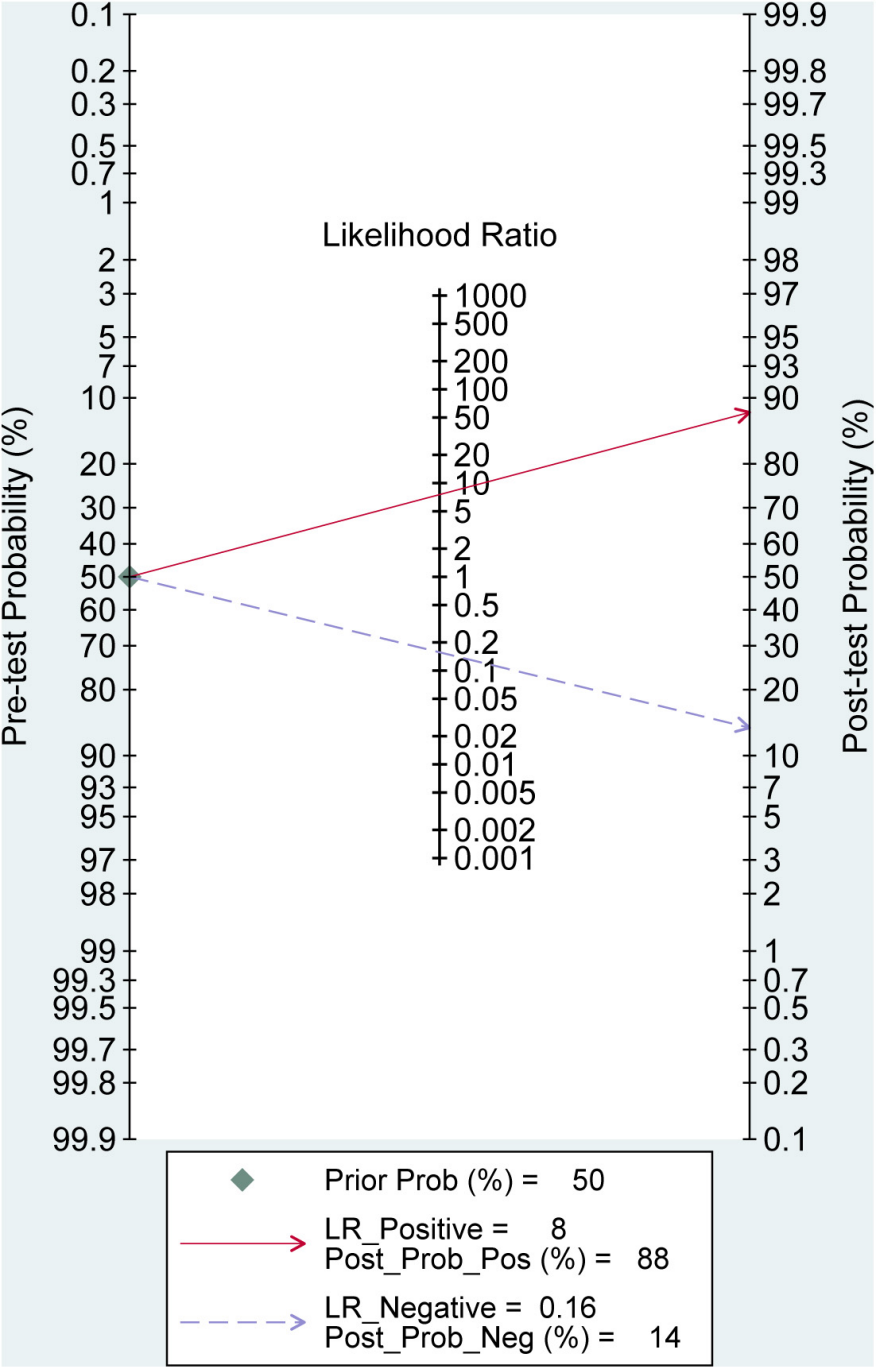
Forest plots of positive likelihood ratio (PLR) and negative likelihood ratio (NLR).

**FIGURE 4:**
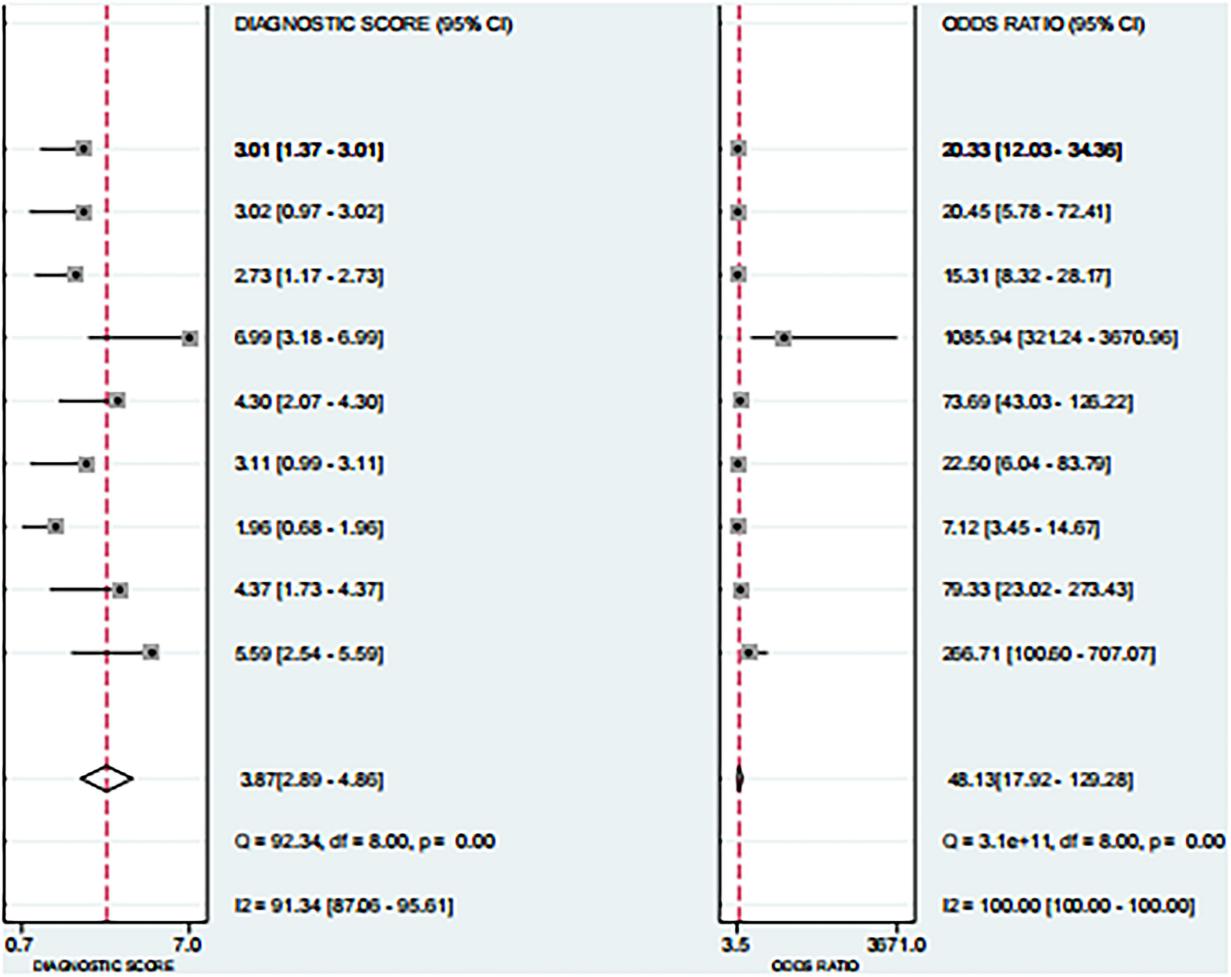
Forest plots of diagnostic odds ratio (DOR) and diagnostic score.

**FIGURE 5:**
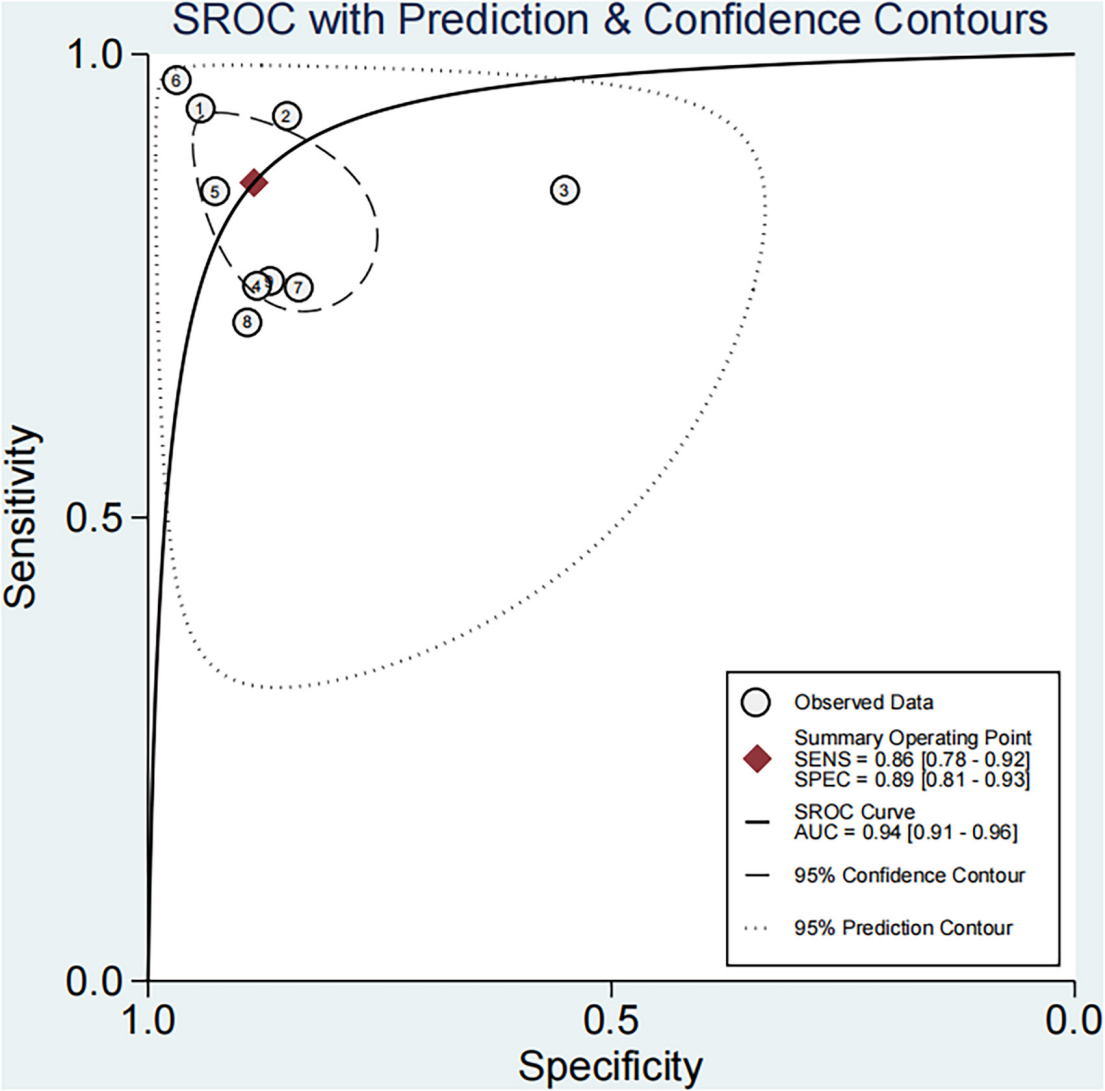
SROC curve for three-dimensional transvaginal ultrasonography in diagnosing intrauterine adhesions.

Based on the nine included studies, the diagnostic performance of three-dimensional transvaginal ultrasonography for intrauterine adhesions was pooled using a bivariate random-effects model. The pooled sensitivity was 0.86 (95% CI: 0.83–0.89), and the pooled specificity was 0.90 (95% CI: 0.87–0.92) (Figure 2). The distribution of sensitivity and specificity across studies was relatively consistent, with most sensitivities ranging from 0.74 to 0.97 and specificities ranging from 0.83 to 0.97. The pooled positive likelihood ratio (PLR) was 8.6 (95% CI: 6.2–11.9), indicating that a positive test result increases the probability of having intrauterine adhesions by more than eightfold compared to the pre-test probability. The pooled negative likelihood ratio (NLR) was 0.16 (95% CI: 0.13–0.20), suggesting that a negative test result reduces the probability of disease to approximately 16% of the pre-test probability (Figure 3). The pooled diagnostic odds ratio (DOR) was 53.2 (95% CI: 34.7–81.4), demonstrating a strong discriminatory ability of the test to distinguish between diseased and non-diseased individuals (Figure 4). The summary receiver operating characteristic (SROC) analysis showed an area under the curve (AUC) of 0.94 (95% CI: 0.91–0.96), indicating high overall diagnostic accuracy (Figure 5). The relatively small areas of the 95% confidence ellipse and prediction ellipse in the HSROC plot suggest moderate between-study heterogeneity. Overall, these results support the use of three-dimensional transvaginal ultrasonography as a reliable imaging modality for the diagnosis of intrauterine adhesions.

### 3.5 Sensitivity analyses

Excluding the single study judged high risk of bias in the QUADAS-2 assessment did not materially change the pooled sensitivity, specificity, PLR/NLR, DOR, or HSROC AUC; all estimates remained within the 95% confidence intervals of the primary analysis. Leave-one-out analyses yielded similarly stable results, indicating that no individual study unduly influenced the summary measures.

### 3.6 Publication bias assessment

**FIGURE 6:**
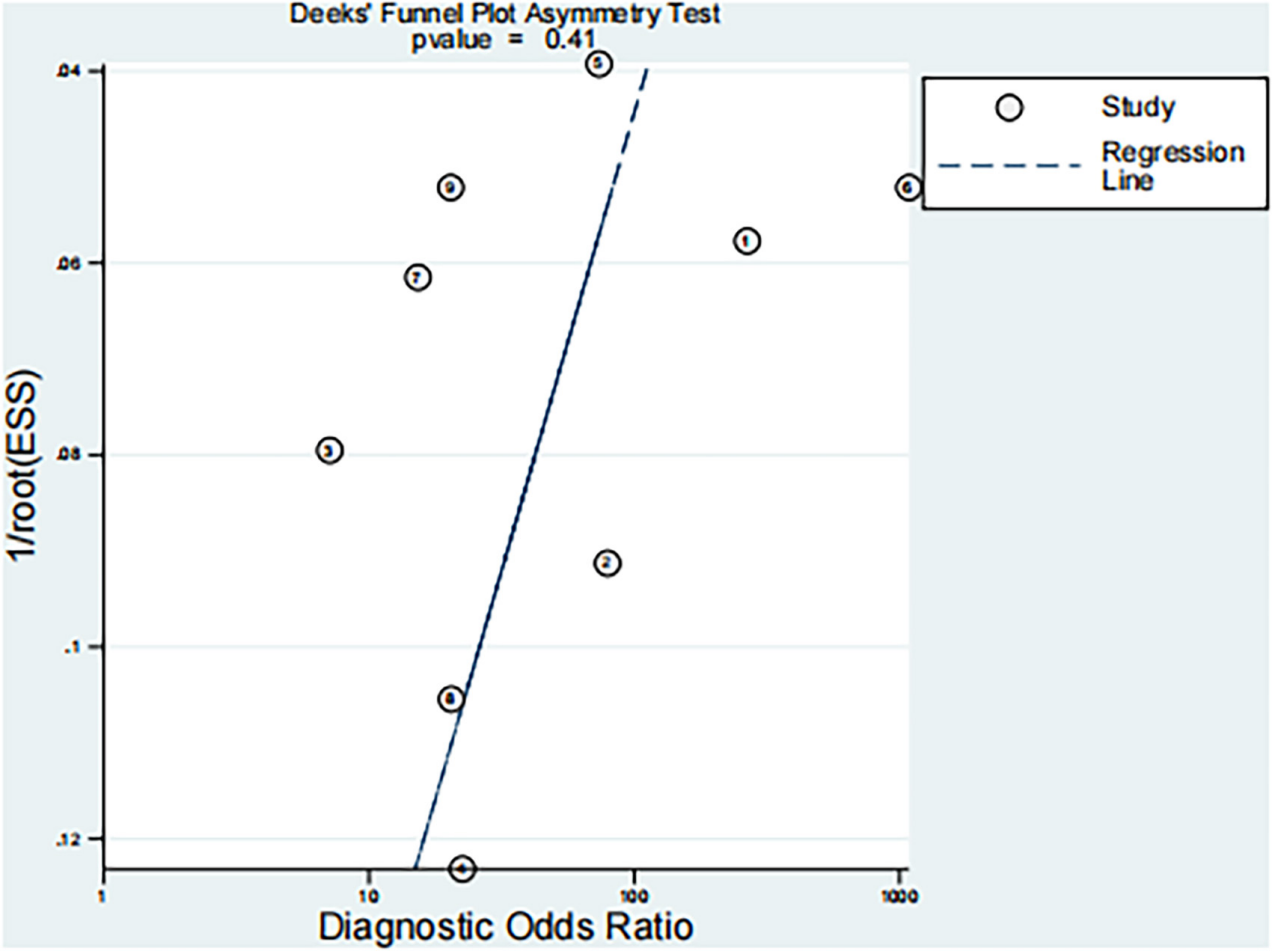
Deeks’ funnel plot with regression test for asymmetry.

Deeks’ funnel plot asymmetry test was performed to evaluate potential publication bias in the included studies based on diagnostic odds ratios (DORs). The regression test yielded a *p*-value of 0.41 (Figure 6), indicating no significant evidence of publication bias.

### 3.7 Clinical utility

**FIGURE 7:**
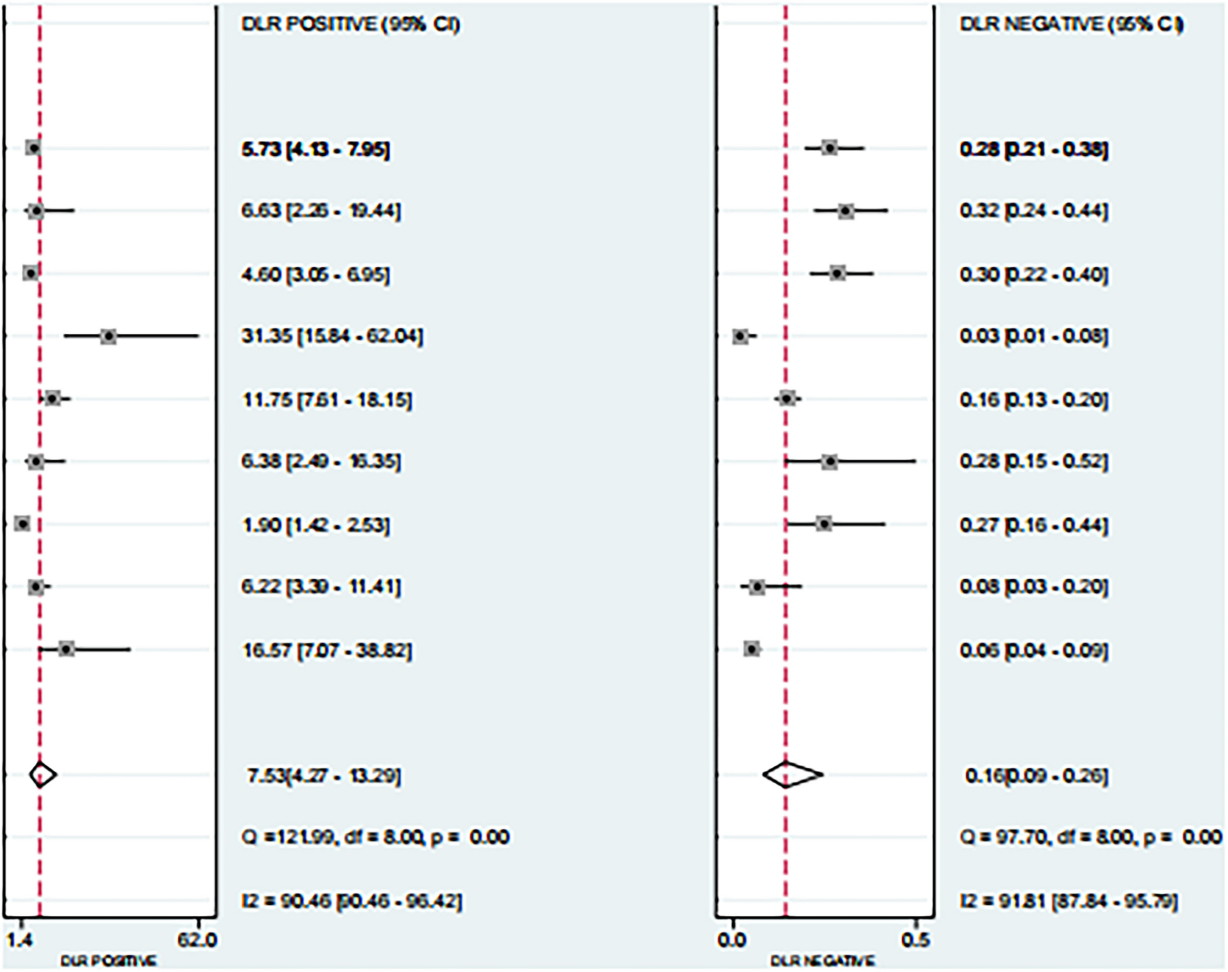
Fagan nomogram for evaluating the clinical utility of 3D transvaginal ultrasound in diagnosing intrauterine adhesions.

The Fagan nomogram analysis demonstrated that, assuming a pre-test probability of 50%, a positive 3D-TVUS result (positive likelihood ratio, LR^+^ = 8) increased the post-test probability of intrauterine adhesions to 88%, whereas a negative result (negative likelihood ratio, LR^+^ = 0.16) reduced the post-test probability to 14% (Figure 7). These findings highlight the strong clinical applicability of 3D-TVUS in both confirming and excluding the diagnosis of IUA in suspected patients.

## 4 Discussion

This meta-analysis synthesized evidence from nine diagnostic accuracy studies to evaluate the performance of 3D-TVUS in diagnosing IUA. The pooled results demonstrated a sensitivity of 0.86, specificity of 0.90, and an AUC of 0.94, indicating that 3D-TVUS is a highly reliable diagnostic tool. These findings suggest that 3D-TVUS can provide accurate preoperative assessment, which is crucial for guiding clinical decision-making and treatment planning in patients with suspected IUA. The high diagnostic performance observed in our analysis can be attributed to the technical advantages of 3D-TVUS. Compared with conventional two-dimensional ultrasonography, 3D-TVUS offers multiplanar reconstruction and volumetric rendering, enabling comprehensive visualization of uterine cavity morphology and endometrial contour (13, 22). This allows for the detection of subtle intrauterine structural abnormalities, such as thin filmy adhesions or irregular endometrial surfaces, which may be missed by traditional imaging (19). Additionally, Doppler assessment incorporated into 3D-TVUS provides functional information on endometrial and myometrial vascularity, potentially improving diagnostic confidence, especially in differentiating IUA from other intrauterine pathologies such as endometrial polyps or submucosal fibroids (28).

When compared with other diagnostic modalities, hysteroscopy remains the gold standard for IUA diagnosis because it allows direct visualization and simultaneous treatment. However, it is invasive, requires anesthesia in some cases, and carries risks of uterine perforation, infection, and adhesion recurrence. Hysterosalpingography (HSG) and sonohysterography (SHG) can also provide diagnostic information but are limited by radiation exposure (HSG), patient discomfort, and reduced specificity in complex cases (7, 29).

Notably, saline infusion sonography (SIS)—especially when combined with 3D technology (3D-SIS)—offers superior visualization compared to 3D-TVUS alone. Bingol et al. (4) directly compared the diagnostic accuracy of SIS, transvaginal ultrasonography (including 3D mode), and hysteroscopy, demonstrating that SIS had a significantly higher sensitivity (92.3% vs. 76.9%) for detecting intrauterine adhesions and concurrent lesions (e.g., endometrial polyps), and could more reliably assess uterine cavity distensibility—a key feature for distinguishing mild adhesions from normal endometrial folds (4). Similarly, Sabry et al. (5) confirmed that 3D-SIS enhanced the delineation of adhesion bands and cavity contour via volumetric reconstruction, reducing the rate of missed mild or focal adhesions that are often overlooked by 3D-TVUS alone (5). However, SIS still requires transvaginal catheterization, which may cause mild discomfort (e.g., cramping) in some patients; thus, 3D-TVUS remains the preferred first-line tool for initial screening, while 3D-SIS can be used as a second-step modality for ambiguous cases (e.g., 3D-TVUS showing unclear endometrial contour or suspected mild adhesions). In this context, 3D-TVUS offers a non-invasive, well-tolerated, and widely available alternative that balances diagnostic accuracy with patient safety (22, 24). Despite its advantages, heterogeneity among the included studies should be acknowledged. Possible sources include variation in study design, operator experience, ultrasound equipment, diagnostic criteria for IUA, and patient characteristics. While the SROC plot suggested only moderate heterogeneity, these factors may still influence real-world applicability. Moreover, the majority of included studies were conducted in single-center Chinese populations, potentially limiting the generalizability of the findings to broader clinical settings. Future multicenter studies involving diverse populations are warranted to validate the external applicability of 3D-TVUS for IUA diagnosis.

From a clinical perspective, the high PLR (8.6) and low NLR (0.16) observed in our analysis indicate that 3D-TVUS can substantially alter post-test probabilities, as confirmed by Fagan’s nomogram. This suggests that a positive result can strongly confirm the presence of IUA, whereas a negative result can effectively rule it out in many cases. Such diagnostic confidence may help avoid unnecessary invasive procedures in patients with low pre-test probability and prioritize hysteroscopic intervention for those with high suspicion.

From a healthcare economics perspective, 3D-TVUS can reduce unnecessary hysteroscopic procedures: for patients with a pre-test probability of IUA < 30%, a negative 3D-TVUS result (NLR = 0.16) reduces the post-test probability to < 5%, avoiding invasive hysteroscopy and its associated costs (e.g., anesthesia, surgical facilities) and risks (e.g., uterine perforation, infection). This is further supported by Burjoo et al. (30), who found that preoperative 3D-TVUS could provide detailed intrauterine anatomical information (e.g., adhesion location, extent) for hysteroscopic adhesiolysis, helping surgeons formulate precise operative plans and reduce intraoperative exploration time—thus indirectly lowering procedure-related costs and complication risks (30). In contrast, a positive 3D-TVUS result (PLR = 8.6) can prioritize patients for hysteroscopic adhesiolysis: by identifying high-probability IUA cases in advance, clinical workflows are optimized, and the waste of surgical resources on patients with low IUA likelihood is avoided, which is particularly valuable in resource-constrained settings.

However, 3D-TVUS requires compatible probes, volumetric acquisition modules, and post-processing software, which may increase upfront costs and training needs compared with conventional 2D ultrasound. Consequently, adoption can be uneven across settings, particularly where capital budgets are constrained. Importantly, in infertility management and ART programs, diagnostic/operative hysteroscopy remains fundamental to excluding concomitant intrauterine pathologies (e.g., synechiae, polyps, submucosal fibroids) prior to embryo transfer. Within such pathways, 3D-TVUS should be viewed as a non-invasive triage and surveillance tool—to screen, to guide timing and necessity of hysteroscopy, and to monitor postoperative cavities—rather than as a replacement for hysteroscopy when therapeutic intervention is needed.

This review has limitations. First, heterogeneity across studies, pertaining to patient spectrum, operator expertise, ultrasound platforms, acquisition and reconstruction protocols, and non-uniform diagnostic thresholds—may influence generalizability. Second, several analyses relied on 2 × 2 data derived from reported metrics, which can introduce classification imprecision. Third, most included cohorts were single-center studies from limited geographic regions, potentially limiting external validity. Fourth, we could not conduct informative subgroup meta-analyses (e.g., by adhesion severity, use of adjunctive 3D-SIS, or Doppler criteria) due to the small number of studies and inconsistent reporting. Finally, publication bias cannot be fully excluded despite a non-significant Deeks’ test.

## 5 Conclusion

Three-dimensional transvaginal ultrasound demonstrates high diagnostic accuracy for detecting intrauterine adhesions when benchmarked against hysteroscopy, with pooled sensitivity ∼0.86, specificity ∼0.90, and an HSROC AUC ∼0.94. These findings support 3D-TVUS as a reliable, non-invasive first-line imaging option for screening and follow-up, particularly where access to operative hysteroscopy is limited. Nevertheless, hysteroscopy remains essential for definitive diagnosis and adhesiolysis in infertility and operative pathways. Future multicenter prospective studies employing standardized acquisition/interpretation protocols and reporting across adhesion severity strata are warranted to refine patient selection and optimize clinical implementation.
